# Plasminogen initiates and potentiates the healing of acute and chronic tympanic membrane perforations in mice

**DOI:** 10.1186/1479-5876-12-5

**Published:** 2014-01-07

**Authors:** Yue Shen, Yongzhi Guo, Malgorzata Wilczynska, Jinan Li, Sten Hellström, Tor Ny

**Affiliations:** 1 Department of Medical Biochemistry and Biophysics, Umeå University, SE-901 87 Umeå, Sweden; 2 Industrial Doctoral School, Umeå University, SE-901 87 Umeå, Sweden; 3 Department of Audiology and Neurotology, Karolinska University Hospital, SE-17176 Stockholm, Sweden

**Keywords:** Plasminogen, Wound healing, Tympanic membrane perforations

## Abstract

**Background:**

Most tympanic membrane (TM) perforations heal spontaneously, but approximately 10-20% remain open as chronic TM perforations. Chronic perforations can lead to an impaired hearing ability and recurrent middle ear infections. Traditionally, these perforations must be surgically closed, which is costly and time consuming. Therefore, there is a need for simpler therapeutic strategies. Previous studies by us have shown that plasminogen (plg) is a potent pro-inflammatory regulator that accelerates cutaneous wound healing in mice. We have also shown that the healing of TM perforations is completely arrested in plg-deficient (plg^-/-^) mice and that these mice develop chronic TM perforations. In the present study, we investigated the therapeutic potential of local plg injection in acute and chronic TM perforation mice models.

**Methods:**

Plg^-/-^ mice and wild-type mice were subjected to standardized TM perforations followed by local injection of plg into the soft tissue surrounding the TM. TM perforations with chronic characteristics were induced by leaving TM perforations in plg^-/-^ mice untreated for 9 days before treatment. The healing process was observed through otomicroscope and finally confirmed by immunostaining. The quality of TM healing was evaluated based on the morphology of the TM.

**Result:**

Daily local injections of plg into the soft tissue surrounding the TM restored the ability to heal TM perforations in plg^-/-^ mice in a dose-dependent manner, and potentiated the healing rate and quality in wild-type mice. A single local injection of plg initiated the healing of the chronic-like TM perforations in these mice, resulting in a closed TM with a continuous but rather thick outer keratinocyte layer. However, three plg injections led to a completely healed TM with a thin keratinizing squamous epithelium covering a connective tissue layer.

**Conclusion:**

Our data suggests that plg is a promising drug candidate for the treatment of chronic TM perforations in humans.

## Background

Tympanic membrane (TM) perforations have a variety of causes, including trauma, middle ear infection and iatrogenic injury [1-3]. Most acute TM perforations heal spontaneously, but approximately 10-20% do not heal and become chronic [4]. Chronic perforations can lead to impaired hearing and recurrent middle ear infections [5]. To date, myringoplasty, a surgical procedure in which the TM is reconstructed by grafting new tissues in the perforated area, has been the traditional method of repairing chronic perforations [6]. However, the cost of this surgery, and the care and sick leave required after surgery, impose a large burden on both the patients and the healthcare system. Therefore, simpler therapeutic strategies are being investigated. In this context, several studies throughout the last decade have sought to identify molecules that may initiate the healing of TM perforations without surgery [7]. In the present study, we show that the plasma protein plasminogen (plg) is a new drug candidate for the treatment of non-healing TM perforations.

Plg is a zymogen that is produced by the liver and is maintained in the blood at a concentration of 2 μM [8]. Plg is activated to the broad-spectrum protease plasmin by either of two physiological plasminogen activators (PAs): tissue-type PA (tPA) or urokinase-type PA (uPA) [9]. It has been well established that plg, due to its ability to degrade fibrin and extracellular matrix proteins, plays an important role in fibrinolysis and in many tissue-remodeling processes including wound healing [9,10]. In addition, we have recently shown that plg is also a potent pro-inflammatory regulator that accumulates in cutaneous wounds and is essential for wound healing. In plg^-/-^ mice, cutaneous wounds do not heal and become chronic, partially due to a disturbed inflammatory phase. In these mice, wounds can heal completely only after plg supplementation. Importantly, plg administration also enhances the healing of acute cutaneous wounds in wild-type (WT) mice and initiates and allows the complete healing of chronic diabetic wounds in diabetic mice [11].

Earlier studies from our group have shown that the healing of TM perforations is completely arrested in plg^-/-^ mice and that intravenous supplementation of these mice with human plg restored their ability to heal TM perforations [12]. We have also shown that active plasmin is important for TM healing and that uPA, but not tPA, plays a central role in the activation of plg to plasmin [13]. These data suggest that plg is essential for the healing of TM perforations. Our finding that plg is a pro-inflammatory regulator that enhances cutaneous wound healing in WT and diabetic mice suggested that plg supplementation might be a potential treatment for chronic TM perforations. However, the main difference between the healing of TM perforations and the healing of skin wounds is the lack of a fibrin-rich provisional matrix in TM wounds. Therefore, during TM healing keratinocytes do not have to migrate through extracellular matrix beneath the wound crust [12].

In the present study, we used the well-established TM perforation model to test the effect of plg on the efficiency of healing in plg^-/-^ mice and WT mice. Our results showed that local injection of plg into plg^-/-^ mice enhanced healing in a dose-dependent manner. With the optimal plg dosage, the TM healing in plg^-/-^ mice was completely rescued, and the healing rate in WT mice was accelerated. In summary, these data provide a basis for future development of plg as a new drug for the treatment of chronic TM perforations in humans.

## Methods

### Animals

Plg-heterozygous mice with a C57BL/6 background were used to generate wild-type (WT), plg-heterozygous (plg^+/-^) and plg-deficient (plg^-/-^) offspring. The mice were genotyped using a rapid chromogenic assay as described previously, and the genotypes were confirmed using PCR [14]. WT and plg^-/-^ siblings at the age of 6–8 weeks were used for the experiments. All of the research protocols were in compliance with the University of Umeå Ethical Committee for Animal Studies.

### Anesthetizing procedure

The mice were anesthetized by an intraperitoneal injection of a 1:1 mixture of Dormicum® (25 μl, Roche AB, Stockholm, Sweden) and Hypnorm™ (25 μl, Janssen Pharmaceutica, Beerse, Belgium) in 50 μl of sterile water.

### Perforation procedure

The mice received TM perforations as described previously [12]. Briefly, the mice were first anesthetized using the Dormicum and Hypnorm mixture. Under an otomicroscope, the pars tensa of the TM was perforated in the posterior superior quadrant using a myringotomy lancet. To induce TM perforations with chronic characteristics, TM perforations of plg^-/-^ mice were performed as described above, after which the perforations were left untouched for 9 days.

### Local injection procedure

**Figure 1:**
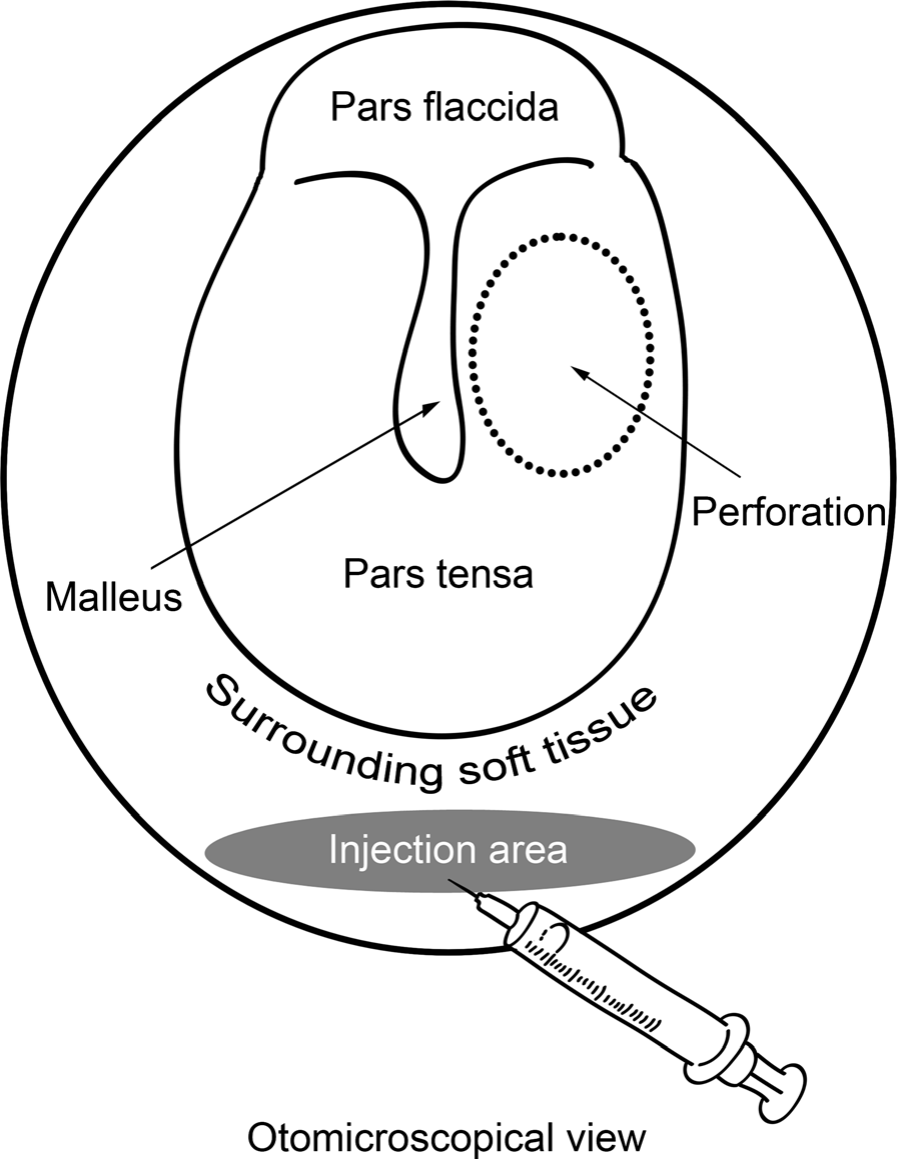
Simplified schematic diagram showing the local injection procedure.

The mice were anesthetized using the Dormicum and Hypnorm mixture. Under an otomicroscope, 20 μl of human plg at different concentrations (Omnio AB, Umeå, Sweden) or phosphate-buffered saline (PBS) was injected into the surrounding soft tissue at the internal end of the external ear canal (shown in Figure 1). The course of healing was observed and documented daily.

### Subcutaneous injection procedure

The mice were anesthetized as described above and then injected at a dorsal subcutaneous site between the back limbs with 100 μl of 10 μg/μl human plg or PBS.

### Intraperitoneal injection procedure

The mice were held by the nape of the neck and extended by pressing the tail to the palm of the hand. Then, 100 μl of 10 μg/μl human plg was injected. The abdominal wall was penetrated in a line parallel with the mouse backbone and at an approximately 45-degree angle to the abdominal wall.

### Preparation of specimens for immunohistochemistry

The mice were first sacrificed, and then the skulls were placed in a 4% buffered formalin solution (pH 7.2) for approximately 1 week. The buffered formalin solution contained 0.0027 M KCl, 0.0015 M KH_2_PO_4_, 0.1369 M NaCl, and 0.0080 M Na_2_HPO_4_. Thereafter, the TMs and surrounding bony rim were dissected out and immersed in phosphate buffer (pH 7.4) overnight. The specimens were then decalcified. Subsequently, they were soaked in phosphate buffer for 1 h. After dehydration in a graded series of ethanol, the specimens were embedded in paraffin. Thin sections (5-μm thick) of the TMs were made using a Leica Microtome (Leica Microsystems AB, Kista, Sweden). The sections were placed on Super Frost Plus glasses slides. Before the staining procedure, the slides were left in a heating cabinet at 37°C overnight.

### Immunohistochemistry

The paraffin-embedded sections were rehydrated and then treated with antibodies as follows: cytokeratin was detected immunohistochemically by the peroxidase anti-peroxidase method using a rabbit anti-human polyclonal antibody (10550, ICN Pharmaceuticals, Aurora, OH) as the primary antibody. In brief, the antigens were first retrieved by treatment with 0.1% trypsin (pH 7.8) at 37°C for 8 min, and then the tissue sections were blocked with 5% non-immunized swine serum (Dakopatts, Sweden), and incubated with the primary antibody, which was diluted 1:100 in PBS. After this procedure, a swine anti-rabbit link antibody (Dako, Glostrup, Denmark) was applied, followed by a rabbit PAP complex (Dako). The staining was visualized through a diaminobenzidine (DAB) reaction (Vector Laboratories, Burlingame, CA) and the sections were counterstained with Mayer’s hematoxylin. The slides were examined by light microscopy under a Leica DMLB microscope, and images were recorded digitally using a Leica DC 300 F camera connected to a personal computer. Adjustment of the contrast and brightness in the individual images was performed using Adobe Photoshop 7.0 software.

### Scoring of TM healing

**Figure 2:**
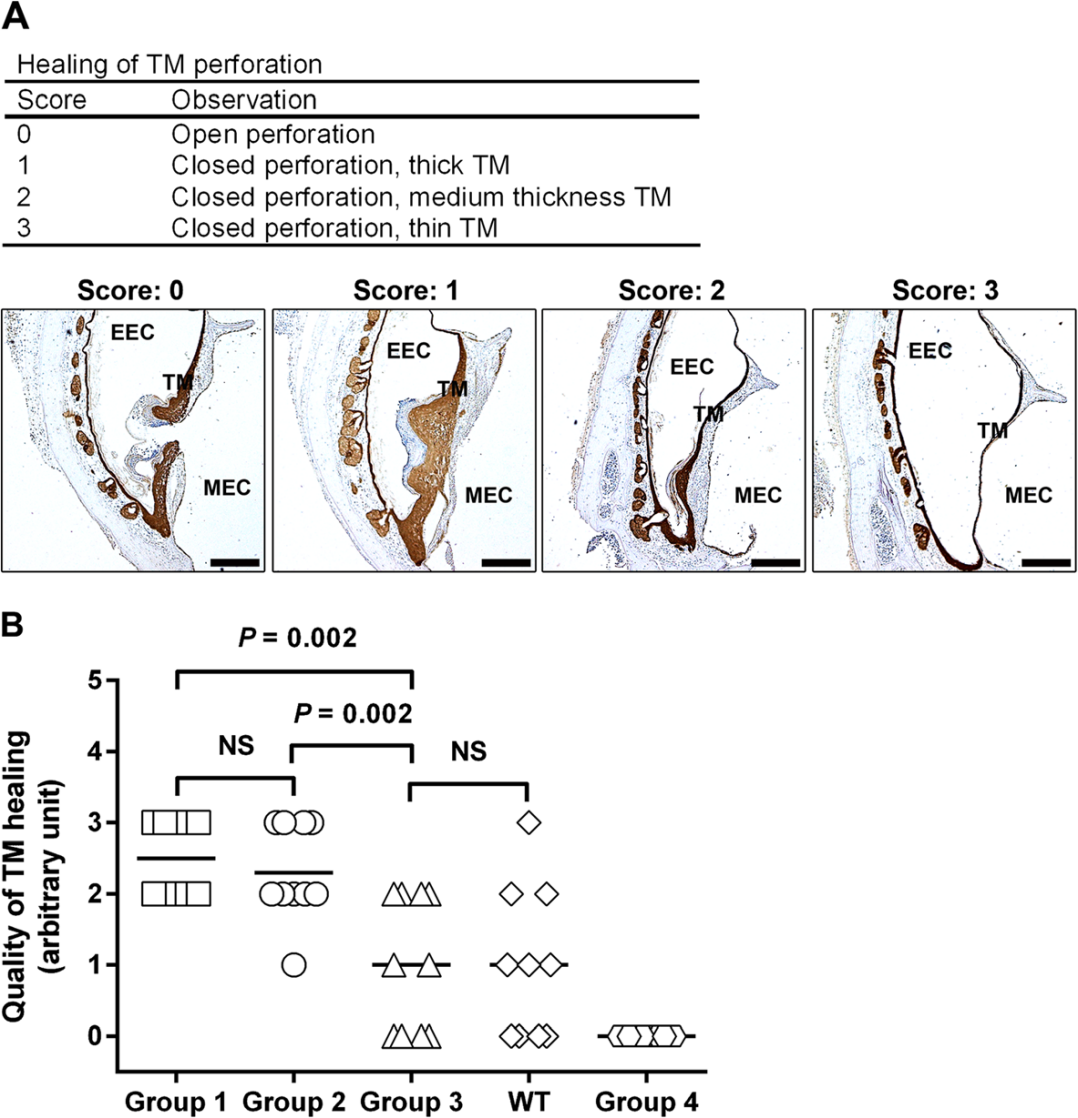
**Characterization of the quality of TM healing in plg**^**-/- **^**mice treated locally with plg. (A)** The scoring system used to evaluate the quality of healing of TM perforations. Scale bar, 400 μm. EEC, external ear canal; MEC, middle ear cavity; TM, tympanic membrane. **(B)** A comparison of quality of TM healing with the mean value marked by a line for mice from Group 1 (plg^-/-^ mice injected with 0.4 mg/day of plg) (squares), Group 2 (plg^-/-^ mice injected with 0.2 mg/day of plg) (open circles), Group 3 (plg^-/-^ mice injected with 0.1 mg/day of plg) (triangles), non-treated WT control mice (diamonds), and Group 4 (plg^-/-^ mice treated with PBS) (hexagons). The quality of healing was evaluated based on IHC keratin staining performed at day 9 after TM perforation. NS indicates not significant. *P* < 0.05 was considered to be significant.

A scoring system was used to evaluate the quality of the healing of the TM perforations. Using the arbitrary scale shown in Figure 2A, the scoring system comprised four levels, from an open perforated TM that is given a score of 0 to a perfectly closed perforation with a thin TM that is given a score of 3. The keratin-stained TM sections were scored by two “blind” investigators.

### Statistical analysis

In Tables comparisons between groups were analyzed by Chi-square test. In Figures the results are expressed as a scatter dot plot. The mean value is indicated as a line. Comparisons between two groups were performed using the Mann–Whitney U-test*. P* < 0.05 was considered to be significant.

## Results

### Local plg injection has a dose-dependent effect on the healing of TM perforations in plg^-/-^ mice

Previous studies by our group have shown that the healing of TM perforations is completely arrested in plg^-/-^ mice, but intravenous administration with plg restores the TM healing ability in these mice [12]. To further investigate the role of plg in the healing of TM perforations, we studied whether local plg injection could also restore the healing of TM perforations in plg^-/-^ mice and if so whether plg stimulated healing in a dose-dependent manner. Plg^-/-^ mice were divided into four groups (Table 1), and standardized TM perforations were made as described in the Methods section and previously [12]. In Groups 1 to 3, the mice were locally injected with 0.4 mg/day, 0.2 mg/day or 0.1 mg/day of plg, respectively, from day 0 to day 8. In Group 4, the mice were locally injected with PBS daily and served as negative controls. In addition, WT mice were subjected to standardized TM perforations and were included as a positive control so that healing in non-treated WT mice could be followed. At day 9, the mice were sacrificed. All of the TM samples were collected and further analyzed by immunohistochemistry (IHC) staining.

**Table 1:** **Dose-dependent healing effect of daily local plg injection in plg**^**-/- **^**mice**

**Treatment group**		**Healed TM perforations/Total TM perforations**
Plg-/- mice	Group 1 (0.4 mg/day)	10/10 (100%)
	Group 2 (0.2 mg/day)	10/10 (100%)
	Group 3 (0.1 mg/day)	6/10 (60%)
	Group 4 (PBS)	0/10 (0%)
WT mice (non-treated)		6/10 (60%)

Data were analyzed by IHC staining at day 9 after the perforations. The healing rate was calculated and is shown in parentheses.

The formation of a continuous keratinocyte layer is the hallmark of a healed TM perforation [15]. We therefore used keratin immunostaining to examine the healing of TM perforation in these mice. As shown in Table 1, all of the TM perforations healed in Group 1 and Group 2 (which received 0.4 mg/day and 0.2 mg/day of plg, respectively), and 60% of the perforations were healed in Group 3 (which received 0.1 mg/day of plg). Consistent with our previous findings [12,13], all of the perforations were open in the PBS-treated group, Group 4. In the non-treated WT control group, 60% of the perforations were healed. Newly healed TM perforations are thicker than normal TMs, and these TMs gradually get thinner over time and revert to the typical appearance [16]. The statistical analysis in Table 2 showed that the differences of healing rates between different doses were statistically significant. The thickness of the TM is a crucial determinant in assessing the quality of TM healing [17]. We therefore used a scoring system to evaluate the quality of TM healing based on IHC analysis. The scoring system is explained in Figure 2A. As shown in Figure 2B, there was no significant difference in the quality of healing between Groups 1 and 2, which received the highest daily doses, but there was a significant difference in the quality of healing between Groups 2 and 3. The quality of healing in plg^-/-^ mice treated with the lowest dose of plg (Group 3) was comparable to that of the non-treated WT control group. As shown previously [12], all of the TMs in the PBS-treated controls (Group 4) remained open and were therefore given a score of 0. These results indicated that local plg injection restored the healing ability in plg^-/-^ mice and that plg treatment exerted a dose-dependent healing effect. The higher plg dosage not only led to a higher healing rate but also resulted in a better quality of healing.

**Table 2:** **Statistical analysis of daily local plg injection in plg**^**-/- **^**mice**

**Multiple comparisons**	***P *** **value**
Group 1 vs. Group 2	N/A
Group 1 vs. Group 3	0.025347^$^
Group 1 vs. Group 4	< 0.0001^$$$^
Group 1 vs. WT mice (non-treated)	0.025347^$^
Group 2 vs. Group 3	0.025347^$^
Group 2 vs. Group 4	< 0.0001^$$$^
Group 2 vs. WT mice (non-treated)	0.025347^$^
Group 3 vs. Group 4	0.003415^$^
Group 3 vs. WT mice (non-treated)	N/A
Group 4 vs. WT mice (non-treated)	0.003415^$^

Data were analyzed at day 9 after the perforations. ^$^*P* < 0.05;^$$^*P* < 0.001; ^$$$^*P* < 0.0001.

### Local plg injection accelerates the healing of acute TM perforations in WT mice

**Figure 3:**
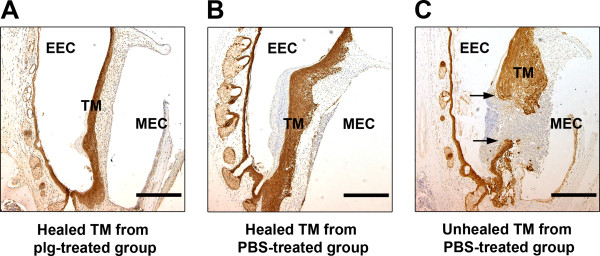
**Keratin staining of TMs in WT mice treated locally with plg or PBS for 7 days. (A)** A representative section of a healed TM from the plg-treated group; **(B)** A representative section of a healed TM from the PBS-treated group; **(C)** An otomicroscopically closed but unhealed TM perforation from the PBS-treated group. The arrows indicate the perforation areas. Scale bar, 200 μm. EEC, external ear canal; MEC, middle ear cavity; TM, tympanic membrane.

**Figure 4:**
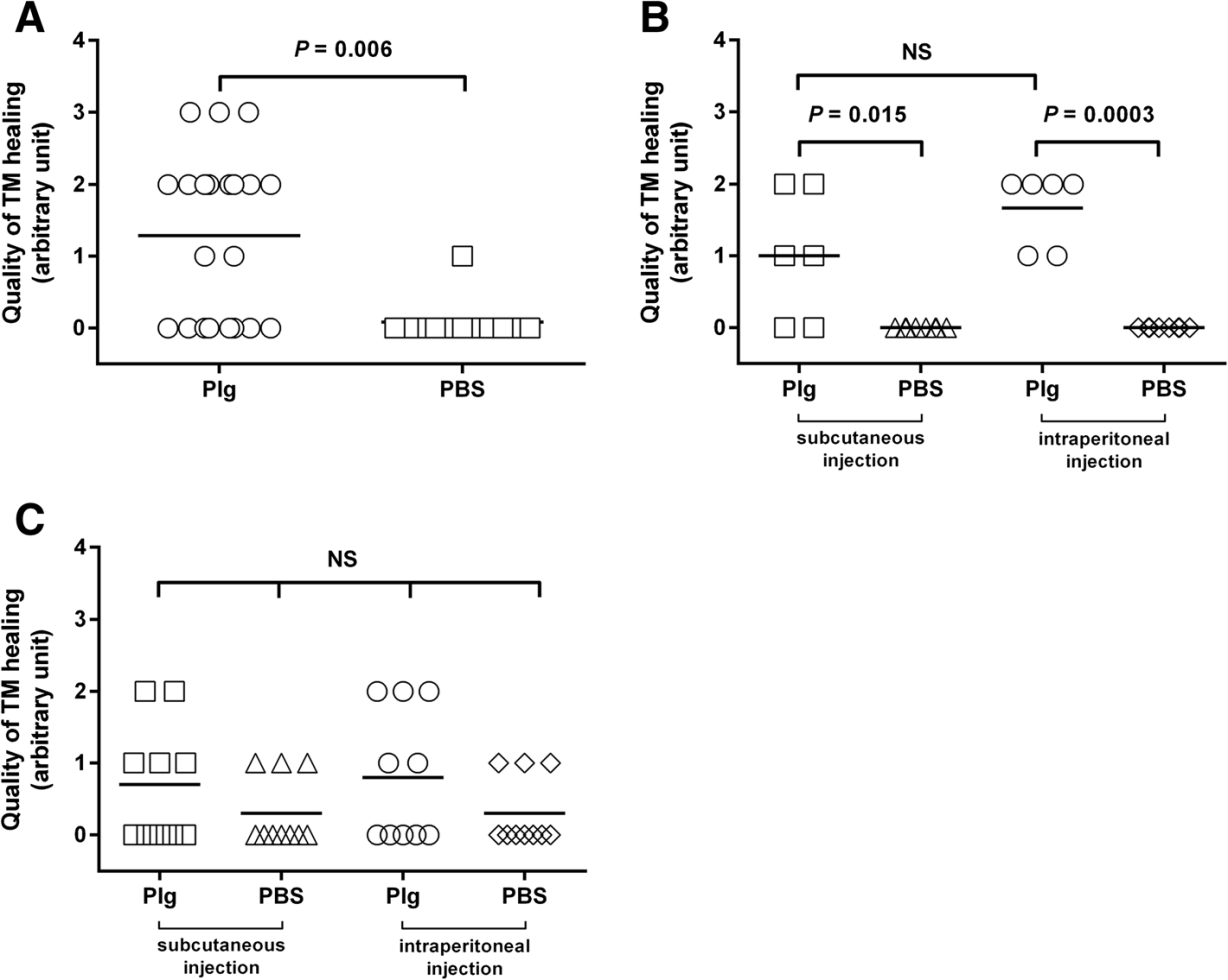
**Characterization of the quality of TM healing in mice. (A)** Comparison of the quality of TM healing of the locally plg-treated group (circles) and the locally PBS-treated group (squares) at day 7 after perforation in WT mice. **(B)** Comparison of the quality of TM healing of the subcutaneous plg-injection group (squares), the subcutaneous PBS-injection group (triangles), the intraperitoneal plg-injection group (circles) and the intraperitoneal PBS-injection group (diamonds) at day 9 after perforation in plg^-/-^ mice. **(C)** Comparison of the quality of TM healing of the subcutaneous plg-injection group (squares), the subcutaneous PBS-injection group (triangles), the intraperitoneal plg-injection group (circles) and the intraperitoneal PBS-injection group (diamonds) at day 8 after perforation in WT mice. NS indicates not significant. *P* < 0.05 was considered to be significant.

The data presented in the previous section showed that plg^-/-^ mice treated with an optimal plg dosage had an even higher healing rate and a better quality of healing than did non-treated WT mice. Additionally, our studies of skin wound healing indicate that plg treatment significantly improved the healing of skin wounds in WT mice [11]. We therefore investigated whether plg treatment has an effect on the healing of acute TM perforations in WT mice. WT mice were subjected to standardized TM perforations and thereafter were locally injected with 0.2 mg/day of plg or PBS from day 0 to day 6. The TM perforations were analyzed daily by otomicroscopy. At day 7, all TM samples were collected, prepared for IHC and analyzed following keratin staining. As shown in Table 3, the daily otomicroscopical analysis revealed that there was a clear difference in the healing patterns of the plg-treated group and the PBS-treated group. At day 5 after perforation, otomicroscopical observation indicated that 14% of the perforations in the plg-treated group were closed, whereas all perforations in the PBS-treated group appeared to be open. At day 7 after perforation, otomicroscopical observation indicated that 62% of the perforations in the plg-treated group were closed, whereas only 25% of the perforations appeared to be closed in the PBS-treated group. IHC keratin staining of TM samples taken at day 7 for keratin revealed that all of the otomicroscopically closed perforations in the plg-treated group were truly healed. The majority of the healed TMs in the plg-treated group also were relatively thin, with a thin outer keratinocyte layer and a rather condensed connective tissue layer with fibroblasts starting to reorganize (Figure 3A). In contrast, only 8% of the perforations in the PBS-treated group were healed, and the only healed TM was still very thick with a massive layer of keratinizing squamous epithelium and a thick connective tissue layer with less well orientated fibroblasts (Figure 3B). The other perforations in the PBS-treated group, which appeared to be otomicroscopically closed, were still open and were found to be covered with inflammatory cells and tissue elements when analyzed using keratin staining (Figure 3C). The quality of TM healing in the WT mice was further evaluated using the scoring system described above. Our results showed that plg treatment resulted in a significantly better quality of healing than did PBS treatment (Figure 4A). In summary, these results indicated that local plg injection accelerated the healing rate and improved the healing quality of acute TM perforations in WT mice.

**Table 3:** Otomicroscopical and IHC analysis of local plg injection treatment for TM perforations in WT mice

**Treatment**	**Otomicroscopical analysis**				**IHC analysis**
	**Day 4**	**Day 5**	**Day 6**	**Day 7**	**Day 7**
Plg-treated	0/21	3/21	8/21	13/21	13/21
	(0%)	(14%)	(38%)	(62%)	(62%)
PBS-treated	0/12	0/12	1/12	3/12	1/12
	(0%)	(0%)	(8%)	(25%)	(8%)
*P* value	N/A	0.169686	0.064794	0.041292^$^	0.002741^$^

The closure rate was calculated and is shown in parentheses. ^$^*P* < 0.05.

### Subcutaneous and intraperitoneal plg injections accelerate the healing of acute TM perforations in mice

It has been shown that after injury, plg is bound to inflammatory cells and are transported to the wound site, which leads to the accelerated healing of wounds [11]. It is therefore likely that non-local administration methods such as subcutaneous and intraperitoneal injections may also affect wound healing. To test this hypothesis, plg^-/-^ mice were subjected to standardized TM perforations and then either injected subcutaneously at a dorsal site between the back limbs or injected intraperitoneally with 1 mg/day of plg or PBS from day 0 to day 8. At day 9, all of the TM samples were collected, prepared for IHC and analyzed following keratin staining. As shown in Table 4, the healing rates in both subcutaneous plg injection group and intraperitoneal plg injection group were significantly faster than their PBS control groups. The healing quality of plg treated groups was also significantly better than their PBS controls (Figure 4B). WT mice were examined in a similar way. The mice were subjected to standardized TM perforations and either injected subcutaneously or injected intraperitoneally with 1 mg/day of plg or PBS from day 0 to day 7. TM samples were collected at day 8. Even though statistical analysis in WT mice failed to show significant differences between plg-treated groups and PBS-treated groups, the tendencies of faster healing and better healing quality in plg-treated groups are observed (Table 5 and Figure 4C).

**Table 4:** **IHC analysis of subcutaneous or intraperitoneal plg injection treatment for TM perforations in plg**^**-/- **^**mice**

**Treatment group**	**Healed TM perforations/Total TM perforations**
Subcutaneous PBS injection	0/8 (0%)
Subcutaneous plg injection	4/6 (67%)
*P* value	0.006285^$^
Intraperitoneal PBS injection	0/8 (0%)
Intraperitoneal plg injection	6/6 (100%)
*P* value	0.000183^$$^

Data were analyzed at day 9 after the perforations. The healing rate was calculated and is shown in parentheses. ^$^*P* < 0.05; ^$$^*P* < 0.001.

**Table 5:** IHC analysis of subcutaneous or intraperitoneal plg injection treatment for TM perforations in WT mice

**Treatment group**	**Healed TM perforations/Total TM perforations**
Subcutaneous PBS injection	3/10 (30%)
Subcutaneous plg injection	5/10 (50%)
*P* value	0.36131
Intraperitoneal PBS injection	3/10 (30%)
Intraperitoneal plg injection	5/10 (50%)
*P* value	0.36131

Data were analyzed at day 8 after the perforations. The healing rate was calculated and is shown in parentheses.

### Local plg injection initiates and allows the complete healing of chronic TM perforations in plg^-/-^ mice

To investigate whether local plg injection can initiate healing of the chronic TM perforations in plg^-/-^ mice, the TM perforation model was used. Plg^-/-^ mice were subjected to standardized TM perforations on day 0 and left untreated from day 1 to day 9 for the development of a chronic type of perforation. As shown previously, these TM perforations are characterized by high neutrophil infiltration, fibrin and necrotic tissue deposition, and arrested keratinocyte migration ([12] and data not shown). On day 10, the mice were divided into three groups (Table 6). The Group 1 mice were locally injected with plg (0.2 mg/day) on days 10, 11 and 12 and left untreated until the end of the experiment. The Group 2 mice were given a single plg injection (0.2 mg) on day 10 and left untreated until the end of the experiment. The Group 3 mice were locally injected with PBS on days 10, 11 and 12 and left untreated until the end of the experiment. At day 19, all of the TM samples were collected and analyzed following keratin staining.

**Table 6:** **IHC analysis of local plg injection treatment for chronic-like TM perforations in plg**^**-/- **^**mice**

**Treatment group**	**Healed TM perforations/Total TM perforations**
Group 1 (plg injections on day 10, 11 and 12)	3/6 (50%)
Group 2 (plg injection on day 10)	2/6 (33%)
Group 3 (PBS injections on day 10, 11 and 12)	0/12 (0%)

Data were analyzed at day 19 after the perforations. The healing rate was calculated and is shown in parentheses.

**Figure 5:**
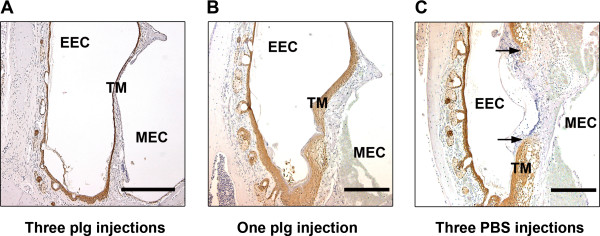
**Keratin staining of chronic-like TM perforations in plg**^**-/- **^**mice treated locally with plg.** TM perforations in plg^-/-^ mice were left untreated for 9 days to acquire the characteristics of chronic wounds. From day 10, plg was locally injected one or three times. The healing of the perforations was analyzed by IHC at day 19. **(A)** A representative section of a chronic TM perforation treated with three plg injections on days 10, 11, and 12 (Group 1); **(B)** A representative section of a chronic TM perforation treated with a single plg injection on day 10 (Group 2); **(C)** A representative section of a chronic TM perforation treated with three PBS injections (Group 3). The arrows indicate the perforated areas. Scale bar, 200 μm. EEC, external ear canal; MEC, middle ear cavity; TM, tympanic membrane.

As shown in Table 6, three plg injections (Group 1) led to 50% of the perforations being healed by day 19, and a single plg injection (Group 2) resulted in approximately 33% of the perforations being healed by day 19. None of the perforations in the PBS treated mice (Group 3) had healed by day 19. The statistical analysis in Table 7 showed that the healing rates in both Group 1 and Group 2 are significantly faster than that in Group 3. As shown in Figure 5, the TMs were obviously thinner in the mice treated with three injections (Group 1) (Figure 5A) as compared to those injected only once (Group 2) (Figure 5B). In the control group (Group 3) treated with PBS, all of the TM perforations were open, and the perforation borders were thick (Figure 5C). These data indicated that even a single plg injection initiated the healing of chronic TM perforations resulting in a closed TM with a continuous but rather thick outer keratinocyte layer. However, for better healing of TM perforations and to obtain good TM morphology, multiple plg injections are required.

**Table 7:** **Statistical analysis of local plg injection treatment for chronic-like TM perforations in plg**^**-/- **^**mice**

**Multiple comparisons**	***P *** **value**
Group 1 vs. Group 2	0.558185
Group 1 vs. Group 3	0.00729^$^
Group 2 vs. Group 3	0.0339^$^

Data were analyzed at day 19 after the perforations. ^$^*P* < 0.05.

## Discussion

In the present study, we demonstrated that local plg injection restored the healing of TM perforations in plg^-/-^ mice in a dose-dependent manner and enhanced the rate and quality of healing of acute TM perforations in WT mice. We also showed that the effect of plg seems to be independent of the administration method. When chronic TM perforations are induced in plg^-/-^ mice, a single plg injection can initiate its healing, resulting in the closure of the keratinizing squamous epithelium and the growth of connective tissue. However, three plg injections led to complete TM healing in which the in-growth of connective tissue was complete and the epithelium had thinned out (Figure 5).

TM perforation is a common condition that affects approximately 3% of the US population and 1% of the population worldwide [18]. The majority of these perforations heal spontaneously; however, approximately 10-20% of these perforations do not heal and become chronic [4]. The most common way to treat chronic TM perforations is through surgery. Over the years, various tissues and materials as well as a variety of surgical procedures have been used with different success rates [19,20]. However, the cost of surgery is high and the burden on both the patients and the healthcare system is great. Therefore, it is important to find an alternative treatment. To reduce the cost, surgical risks and hospitalization period, such a treatment should preferably be a non-surgical out-patient procedure.

Our previous studies of wound healing have shown that systemic administration of plg restored the arrested healing of TM perforations in plg^-/-^ mice and accelerated the healing of acute and diabetic skin wounds in mice [11,12]. However, to apply systemic administration in humans would require a large amount of plg and for many reasons not be possible in clinical practice. In the present study, we demonstrated that complete healing of TM perforations could be obtained in plg^-/-^ mice following local injection of plg. To use topical application of plg on TMs would be an even better therapeutic choice. In fact, our preliminary data in plg^-/-^ mice indicated that TM healing can be obtained following topical application of plg (data not shown). In the future, the use of local plg injections or even topical plg applications may therefore become feasible therapeutic choices to treat patients with TM perforations.

We also previously showed that plg bound to cell surface receptors on inflammatory cells are transported to the wound site, where the level of plg is locally increased, which leads to a pro-inflammatory cell activation and accelerated wound healing [11]. It is known that the binding of plg to the surface of inflammatory cells can reach a saturation point when the plg concentration increases [21]. In the present study, we found that the effect of plg on the healing of TM perforation is dose-dependent (Tables 1 and 2, Figure 2). Based on our previous results, it is reasonable to speculate that when a low plg dose is administered, less plg is bound to the cells compared to when a higher dosage is administered, and therefore, less plg is transported to the wound area. The lower dose therefore results in non-optimal activation of inflammatory cells and less pronounced wound healing. When the amount of injected plg is increased, the maximum binding of plg to the cells may be reached, and the cell surface then becomes saturated by plg. This may explain the dose-dependent and saturated healing effect we observed (Table 1 and Figure 2B). Furthermore, because plg is transported to the wound site by inflammatory cells, one can speculate that different modes of plg administration might have similar effects on healing. The results obtained in the present study (Tables 4 and 5, Figure 4B and C) support this conclusion. Thus, even though plg was not injected locally, it still stimulated wound healing in a fashion similar to local injections.

We previously showed that plg^-/-^ mice can be used as a model system to investigate the healing of chronic TM perforations [12]. TM perforations in plg^-/-^ mice cannot heal and acquire the characteristic features of chronic wounds, which include the accumulation of inflammatory cells, the lack of necrotic tissue debridement, and often, bacterial infections [12,22]. In the present study, we showed that even if TM perforations in plg^-/-^ mice have remained open for 10 days, healing could be initiated by a single plg injection (Tables 6 and 7, Figure 5). In plg^-/-^ mice that received a single injection of plg, necrotic tissue was removed, and there was no more excessive inflammatory cell accumulation. The keratinocytes that before the plg injections seemed to lack orientating capacity became re-orientated after a single plg injection and started to migrate to form a closed but thick outer keratinocyte layer resting on a thick layer of loose connective tissue. However, to obtain a healed TM, with a thin outer keratinocyte layer that more closely resembled a normalized TM, at least three plg injections were required. This result suggests that plg is not only important for the initiation and termination of the inflammatory phase in chronic TM perforations but that plg is also important for the tissue remodeling that occurs later during TM healing.

## Conclusions

We have shown that local injection of plg into the soft tissue surrounding the TM restored the healing ability of chronic TM perforations in mice. These data suggest that plg therapy may become a novel treatment for chronic TM perforations to be used in clinical practice.

## Abbreviations

IHC: Immunohistochemistry; PA: Plasminogen activator; PBS: Phosphate-buffered saline; Plg: Plasminogen; Plg+/-: Plasminogen heterozygous; Plg-/-: Plasminogen deficient; TM: Tympanic membrane; tPA: tissue-type plasminogen activator; uPA: urokinase-type plasminogen activator; WT: Wild-type.

## Competing interests

TN, SH and JL have patented the use of plasminogen for the treatment of wound healing. They are also stock holders in a start-up company which owns the right to develop plasminogen for therapeutic purposes.

## Authors’ contributions

YS, YG, JL and TN conceived and designed the experiments. YS and YG performed the experiments. YS, YG, MW, SH and TN analyzed the data. YS, MW, SH and TN wrote and prepared the manuscript. All authors read and approved the final manuscript.
